# The dataset for validation of customer inspiration construct in Malaysian context

**DOI:** 10.1016/j.dib.2019.104131

**Published:** 2019-06-11

**Authors:** Arsalan Mujahid Ghouri, Tai Mei Kin, Nek Kamal bin Yeop Yunus, Pervaiz Akhtar

**Affiliations:** a Universiti Pendidikan Sultan Idris, Malaysia; b University of Hull, UK

## Abstract

This study intended to validate customer inspiration (CI)in Malaysian/developing country context. Data were collected from two different respondents for two studies - from Millennial customers of the auto industry and Generation Z customers of the smartphone industry. The survey conducted through a standardized and structured questionnaire. The variables of the both studies were customer-defined market orientation (MO) (customer orientation, competitor orientation, and interfunctional coordination), CI (inspired-by and inspired-to), and customer loyalty (CL). This research strategy, in terms of quantity, is descriptive and correlational. Statistical analysis of the data was carried out, using ADANCO 2.0. The finding of the study suggests all results of data 1 and data 2 were significant, and CI mediates the sub-constructs of MO with CL.

## 1 Data

The data collected on the following constructs: customer-defined market orientation (CDMO) [1], customer inspiration (CI) [2], and customer loyalty (CL) [3].

### 1.1 Demographic characteristics of respondents

**Fig. 1:**
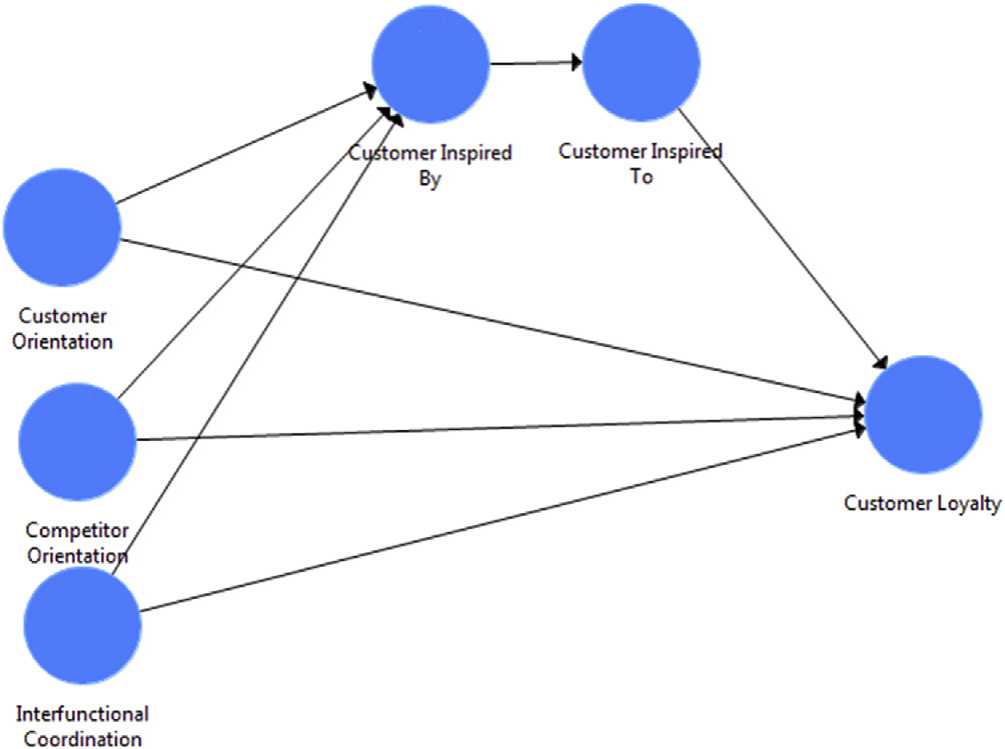
Study model.

In order to verify the construct validation of customer inspiration, the data collected from two generations members – ‘Millennial’ and ‘Generation Z’ in two survey studies (see Fig. 1). The reason to choose Millennial to get response for the auto industry as they reached the age of job/business, therefore, most of them own the vehicle to commute in Malaysia. On the other hand, Generation Z members getting education and living away from their hometown/parents, hence, all respondent had smartphone to communicate with family and friends. The respondents belonged to 11 states of Malaysia. The data consist of 271 responses of Millennial in data 1, and 252 responses of Generation Z in data 2 [4]. recommended that number of respondents should be at least 100 [5]. argued that the number of respondents should be at least 200, and [6] claimed the minimum desirable number of respondents to be 250 [7] offered a rough rating scale for adequate sample sizes in factor analysis: 100 = poor, 200 = fair, 300 = good, 500 = very good, 1000 or more = excellent.

The data collection took 42 days for both studies. The questionnaire was self administrative and in the English language. Data collection adhere all ethical consideration suggested by prominent studies [8], [9]. Table 1, Table 2 illustrate the details of the demographics of respondents of both studies.

**Table 1:** Millennial sample characteristics for study 1 (n = 271).

Category	Description	Numbers	%
Gender	Male	184	67.90
	Female	87	32.10
Education level	Never attended school	0	0
	Attended school	13	4.80
	Diploma	82	30.26
	Degree	129	47.60
	Masters	47	17.34
States and federal territories	Johor DarulTa'zim	3	1.11
	Kedah Darul Aman	2	0.73
	Kelantan DarulNaim	5	1.85
	Malacca	1	0.37
	Pahang	4	1.48
	DarulMakmur Penang	15	5.54
	Perak DarulRidzuan	78	28.78
	Perlis InderaKayangan	2	0.73
	Sabah	8	2.95
	Sarawak	1	0.37
	Selangor Darul Ehsan	77	28.41
	Kuala Lumpur	75	27.68

**Table 2:** Generation Z sample characteristics for study 2 (n = 252).

Category	Description	Numbers	%
Gender	Male	93	36.90
	Female	159	63.40
Education level	Never attended school	0	0
	Attended school	22	8.73
	Diploma	144	57.14
	Degree	86	34.13
States and federal territories	Johor DarulTa'zim	6	2.38
	Kedah Darul Aman	3	1.19
	Kelantan DarulNaim	18	7.14
	Malacca	4	1.59
	Pahang	6	2.38
	DarulMakmur Penang	24	9.52
	Perak DarulRidzuan	67	26.59
	Perlis InderaKayangan	3	1.19
	Sabah	4	1.59
	Sarawak	3	1.19
	Selangor Darul Ehsan	57	22.62
	Terengganu Darul Iman	9	3.57
	Kuala Lumpur	46	18.25
	Putrajaya	2	0.79

**Table:** Specifications Table

Subject area	*Marketing*
More specific subject area	*Customer inspiration, Validation of construct*
Type of data	*Table and text file*
How data was acquired	*Survey method, PLS SEM*
Data format	*filtered, analyzed, descriptive, statistical*
Experimental factors	*Customer loyalty (dependent), customer inspiration (mediator)*
Experimental features	*Data were collected from survey from two different respondents for two studies - from Millennial customers of the auto industry and Generation Z customers of the smartphone industry*
Data source location	*Data gathered from Millennial residents of 13 states, and Generation X from 15 states of Malaysia.*
Data accessibility	*Data provided with the article*
Related research article	*D. Webb, C. Webster, A. Krepapa*[1] *An exploration of the meaning and outcomes of a customer-defined market orientation* *J. Bus. Res., 48 (2000), pp. 101–112.*

**Table:** 

**Value of the data** • This data validates the customer inspiration tool in Malaysian/developing country context. • This data could use for comparison of Millennial and Generation X opinions about customer-defined market orientation, customer inspiration, and customer loyalty with other studies in the field and may part of potential meta-analyses. • The datasets provide information about auto industry and the smartphone industry. • The paper allows other researchers to extend the statistical analysis i.e. ANOVA.

## 2 Experimental design, materials and methods

All items were adopted from reliable studies measure through reflective scale. Table 3 and Table 4 provide the constructs detail, source, coding, loading values, reliability and convergent validity of both studies. Table 5 and Table 6 show the discriminant validity of data 1 and data 2. Furthermore, all items gauge on five-points Likert scale. A PLS-SEM was applied using ADANCO 2.0. Present study model consists of CuO, CoO, and InF (sub-constructs of CDMO), InB and InT (sub-constructs of CI) and CL. All measures were subjected to check the reliability and validity. We employ Jöreskog's rho to check reliability [10]. We adopt convergent validity, with average variance extracted (AVE) and discriminant validity, with the Heterotrait-Monotrait ratio of correlation (HTMT) [10]. The minimum threshold of Jöreskog's rho is more than 0.7, AVE is at most 0.85, and HTMT at least 0.5. All results are delineated evidence for the proposed model constructs, which allow further analysis [11]. For data 1, the Jöreskog's rho value is between 0.8555 and 0.9259, AVE is between 0.5853 and 0.7958, and HTMT correlation is at least 0.5 between all variables. For data 2, the Jöreskog's rho value is between 0.8138 and 0.9275, AVE is between 0.6394 and 0.7984, and HTMT correlation is at least 0.5 between all variables.

**Table 3:** AVE and reliability results and evaluation of the measurement model for study 1.

Construct	Source	Item Coding	Loading	Jöreskog's rho (ρ_c_)	AVE
Customer orientation	[1]			0.8621	0.5853
		CuO1	0.7132		
		CuO2	0.7493		
		CuO3	0.7389		
		CuO4	0.7352		
		CuO5	0.8433		
		CuO6	0.8024		
Competitor orientation	[1]			0.9259	0.7958
		CoO1	0.9282		
		CoO2	0.9175		
Interfunctional coordination	[1]			0.9108	0.7627
		InF1	0.9224		
		InF2	0.8933		
		InF3	0.9379		
Inspired by	[2]			0.8991	0.6241
		InB1	0.7974		
		InB2	0.7230		
		InB3	0.7902		
		InB4	0.8470		
		InB5	0.7556		
		InB6	0.8059		
		InB7	0.8453		
		InB8	0.7523		
		InB9	0.7610		
		InB10	0.8007		
		InB11	0.8162		
		InB12	0.7752		
Inspired to	[2]			0.9071	0.6863
		InT1	0.8977		
		InT2	0.7694		
		InT3	0.9011		
		InT4	0.8607		
		InT5	0.7685		
		InT6	0.7594		
Customer loyalty	[3]			0.8555	0.6279
		CL1	0.7051		
		CL2	0.7965		
		CL3	0.8495		
		CL4	0.7989		
		CL5	0.8048

**Table 4:** AVE and reliability results and evaluation of the measurement model for study 2.

Construct	Source	Item Coding	Loading	Jöreskog's rho (ρ_c_)	AVE
Customer orientation	[1]			0.8138	0.6394
		CuO1	0.7269		
		CuO2	0.8087		
		CuO3	0.7604		
		CuO4	0.7914		
		CuO5	0.8918		
		CuO6	0.8090		
Competitor orientation (CO)	[1]			0.9275	0.7984
		CoO1	0.9106		
		CoO2	0.9381		
Interfunctional coordination	[1]			0.8908	0.7846
		InF1	0.9172		
		InF2	0.8777		
		InF3	0.8659		
Inspired by	[2]			0.8284	0.6582
		InB1	0.8047		
		InB2	0.8496		
		InB3	0.8498		
		InB4	0.8372		
		InB5	0.8220		
		InB6	0.7883		
		InB7	0.8257		
		InB8	0.7506		
		InB9	0.7164		
		InB10	0.8428		
		InB11	0.8299		
		InB12	0.8771		
Inspired to	[2]			0.8471	0.6808
		InT1	0.8795		
		InT2	0.7456		
		InT3	0.8866		
		InT4	0.8854		
		InT5	0.7405		
		InT6	0.7981		
Customer loyalty	[3]			0.8842	0.6817
		CL1	0.8833		
		CL2	0.8620		
		CL3	0.8546		
		CL4	0.7768		
		CL5	0.7425

**Table 5:** Heterotrait-Monotrait ratio of correlation results for study 1.

Construct	Cuo	CoO	InC	InB	InT	CL
Customer orientation (CuO)						
Competitor orientation (CoO)	0.5980					
Interfunctional coordination (InC)	0.5701	0.4594				
Inspired by (InB)	0.7954	0.5563	0.5935			
Inspired to (InT)	0.7925	0.7991	0.5984	0.7209		
Customer loyalty (CL)	0.8209	0.6642	0.6184	0.7607	0.7781

**Table 6:** Heterotrait-Monotrait ratio of correlation results for study 2.

Construct	Cuo	CoO	InC	InB	InT	CL
Customer orientation (CuO)						
Competitor orientation (CoO)	0.6363					
Interfunctional coordination (InC)	0.5725	0.5758				
Inspired by (InB)	0.6144	0.6313	0.5461			
Inspired to (InT)	0.6411	0.8473	0.5351	0.7176		
Customer loyalty (CL)	0.6509	0.7172	0.6409	0.7212	0.7502	

The all direct and indirect relationships were significant, portray in Table 7, Table 8 for both studies. For data 1, Cohen's f^2^ is between 0.1282 (CoO ->InB) to 0.4105 (CoO ->InT), β is between 0.1377 (CoO ->InB) to 0.4927 (CuO ->InB), and t-value is between 1.9597 (InT -> CL) to 8.0484 (CoO ->InB). For data 2, Cohen's f^2^ is between 0.148 (InB -> CL) to 0.4262 (CoO ->InT), β is between 0.1665 (InF ->InT) to 0.5229 (CoO ->InT), and t-value is between 2.288 (InT -> CL) to 6.8271 (CoO ->InT) [12], [13], [14], [15].

**Table 7:** Effect size, direct and indirect effects of the measurement model for study 1.

Effect	Cohen’s f^2^	Direct Effect			Indirect Effect			Total Effect		
		β	Mean	*t*-value	β	Mean	*t*-value	β	Mean	*t*-value
CuO ->InB	0.3334	0.4927	0.4969	8.0484	-	-	-	0.4927	0.4969	8.0484
CuO ->InT	0.2487	0.3653	0.3655	7.7565	-	-	-	0.3653	0.3655	7.7565
CuO -> CL	0.2189	0.3268	0.3240	3.5221	0.1589	0.1626	3.4086	0.4857	0.4855	6.9480
CoO ->InB	0.1282	0.1377	0.1389	2.1229	-	-	-	0.1377	0.1389	2.1229
CoO ->InT	0.4105	0.4506	0.4472	7.3925	-	-	-	0.4506	0.4472	7.3925
CoO -> CL	0.2008	0.1283	0.1343	1.8732	0.0923	0.0893	2.8341	0.2206	0.2235	3.2942
InC ->InB	0.1807	0.2272	0.2228	3.6988	-	-	-	0.2272	0.2228	3.6988
InC ->InT	0.2614	0.1702	0.1722	3.7861	-	-	-	0.1702	0.1722	3.7861
InC -> CL	0.2066	0.1261	0.1242	2.6330	0.0735	0.0737	2.8264	0.1996	0.1979	3.8807
InB -> CL	0.2551	0.2207	0.2262	3.5030	-	-	-	0.2207	0.2262	3.5030
InT -> CL	0.2157	0.1374	0.1329	1.9597	-	-	-	0.1374	0.1329	1.9597

**Table 8:** Effect size, direct and indirect effects of the measurement model for study 2.

Effect	Cohen’s f^2^	Direct Effect			Indirect Effect			Total Effect		
		β	Mean	*t*-value	β	Mean	*t*-value	Β	Mean	*t*-value
CuO ->InB	0.1914	0.2820	0.2819	3.9532	-	-	-			3.9532
CuO ->InT	0.1598	0.1982	0.1990	3.1772	-	-	-	0.1982	0.1990	3.1772
CuO -> CL	0.2337	0.1563	0.1549	2.3858	0.0987	0.1014	2.4399	0.2550	0.2563	4.3923
CoO ->InB	0.1770	0.2557	0.2618	3.0344	-	-	-	0.2557	0.2618	3.0344
CoO ->InT	0.4262	0.5229	0.5234	6.8271	-	-	-	0.5229	0.5234	6.8271
CoO -> CL	0.3248	0.1501	0.1518	2.5822	0.1612	0.1596	2.9655	0.3113	0.3114	3.3294
InC ->InB	0.1774	0.2414	0.2379	3.1996	-	-	-	0.2414	0.2379	3.1996
InC ->InT	0.1487	0.1665	0.1659	2.7640	-	-	-	0.1665	0.1659	2.764
InC -> CL	0.2885	0.2336	0.2313	3.8675	0.0838	0.0841	2.3120	0.3175	0.3154	4.4342
InB -> CL	0.1480	0.2032	0.2109	2.3162	-	-	-	0.2032	0.2109	2.3162
InT -> CL	0.3383	0.2089	0.1991	2.2880	-	-	-	0.2089	0.1991	2.2880

### 2.1 Mediation results

This study tested three sequential mediation results in each of the dataset. In data 1 and 2, the relationships checked are: CuO ->InB ->InT -> CL, CoO ->InB ->InT -> CL, and InF ->InB ->InT -> CL. In data 1, CuO -> CL, CoO -> CL, and InF -> CL relationships is partially mediated by InB ->InT by 32.71%, 41.84%, and 36.82% respectively. In data 2, CuO -> CL, CoO -> CL, and InF -> CL relationships also partially mediated by InB ->InT by 38.81%, 51.78%, and 26.39%. All results are illustrate in Table 7, Table 8.
